# Prioritizing simulation-based stress tests to assess the resilience of transport systems: a computation-free methodology

**DOI:** 10.1186/s43065-025-00128-0

**Published:** 2025-04-17

**Authors:** Hossein Nasrazadani, Maria Nogal, Bryan T. Adey, Stergios A. Mitoulis

**Affiliations:** 1 ETH Zurich, Zurich, Switzerland; 2 TU Delft, Delft, The Netherlands; 3 University of Birmingham, Birmingham, UK

**Keywords:** Resilience, Stress Test, Bootstrapping, Importance sampling, Transportation Systems, Climate Change

## Abstract

This paper introduces a computation-free method for evaluating and prioritizing simulation-based stress tests for resilience assessment of transport systems. It enables infrastructure managers to efficiently screen and rank stress tests, optimizing the selection process to maximize insights into system resilience while minimizing computational demands. Stress tests have been proven to be a practical tool for understanding and mitigating the impact of disruptive events, yet conducting all possible tests using simulations, particularly for complex systems including plausible scenarios to account for climate change and other stressors, is computationally impractical, thus discouraging their use in practice. To address this, the paper suggests a methodology to estimate the impact of stress tests on risks at no computation cost and rank them accordingly to be selected for more detailed assessment. It uses the results of an initial risk assessment and, through a novel implementation of importance sampling and Bootstrapping resampling, selects subsets of the initial results to mimic specific stress test conditions, estimating their impact on risks. The methodology was validated through application to a Swiss road network facing flooding, demonstrating its practical effectiveness in identifying stress tests with significant potential impact on risks, hence having higher priority for more detailed assessment. In the presented case study, the proposed method enabled instant screening of 80 stress test scenarios, saving approximately 56 weeks of computation.

## Introduction

A stress test, in the context of transport systems, is “a set of one or more hypothetical scenarios designed to help determine if a transport system can continue to provide an acceptable level of service when subjected to one or more potentially disruptive events” [2]. Stress testing enables infrastructure managers to conduct scenario-based planning by identifying and conducting scenarios that are of concern and evaluate the performance of the system and risks under those scenarios and if needed, plan and take appropriate measures to achieve satisfactory results. Herein, transport systems include the physical assets, the environment they are embedded in, including hazards and other interdependent systems, and the responsible organization.

Stress testing complements traditional risk assessment by addressing its limitations. While traditional risk assessments focus on estimating the likelihood and consequences of known disruptive scenarios based on historical data, they don’t necessarily highlight the behavior of the system under *stressed* situations, i.e., situations where part(s) of the system are significantly worse than initially designed, planned, or expected, referred to as the *reference* situation [2]. An example of such stressed situations is extreme rainfall events happening more frequently and with greater intensity than planned for, due to climate change. Stress testing has gained significant traction from scholars, practitioners, and regulators [15, 16, 36]. A more detailed review of literature on stress testing is provided subsequently. In essence, the goal of stress testing is to define scenarios that are of most relevance and concern, evaluate risks, and assess whether risks are acceptable or not, in which case adaptation measures are proposed.

Quantitative methods for risk assessment and stress testing, particularly simulation-based approaches allowing for probabilistic risk analysis (PRA), are proven to provide significantly more insights into the uncertainties, and help identify weak assets, links, and operations within the system that might not be recognized by streamlined risk assessments, thus offering clear incentives for practical applications [1, 20, 31, 43]. A simulation-based PRA is often comprised of a set of integrated models each representing part of the system and its uncertainties, thus establishing a virtual twin for the system. Risk assessment is done via stochastic scenario generation, each representing a random realization of the system from hazard occurrence to the ensuing impacts and consequences, concluding with the estimation of risks.

Each part of the system, or more specifically, each variable representing the loading, state of a part of the system, and its recovery, e.g., hazard intensity, structural resistance of assets, or contingency budget for restoration, can be the subject of a stress test. This suggests that there are at least as many potential stress tests as the number of system variables, which can be substantial for complex systems. The number of stress tests can further increase if several stress test scenarios per variable are defined. Therefore, there exists a vast array of potential stress tests, reflecting the multitude of scenarios and complexities that need to be assessed for risk acceptability under various disruption scenarios. However, given the high computational demand for simulation-based assessments, it is not feasible to execute all stress tests, rather, prioritize them and execute simulations only for some. In this paper, prioritization is done through comparing the potential impact of stress tests on risks under similar *levels of stress*. The level of stress, as will be elaborated in the methodology section, depends on the change that is made to the value of the respective variable because of the stress test.

Although the selection of a relatively small but high-priority number of stress tests would result in substantial time savings, literature lacks a computationally efficient method to identify them without necessarily conducting them. The closest approach that one could argue useful is sensitivity analysis (SA). Nevertheless, its application in the context of stress testing has challenges. Current use of SA methods are focused on determining how variations of a variable, considering a given probability distribution function (PDF), impact the output of the system, e.g., risks [40]. A stress test, on the other hand, requires modification of the PDF of the variable, reflecting higher likelihood of more unfavorable values of the variable. Existing SA methods, however, fall short in capturing the impact of such changes without the need for rerunning simulations [37]. This highlights the need to prioritize stress tests before embarking on rerunning the simulations. This limitation is elaborated in the *Need for prioritizing stress tests* section.

To address this gap, this paper proposes, for the first time, a computation-free method that prioritizes simulation-based stress tests for simulation-based assessment. The proposed methodology estimates the impact of stress tests on risks without running simulations and ranks them to be considered for more detailed assessment using simulations. For impact estimation, a novel implementation of bootstrapping and importance sampling approaches is developed that utilizes the results of the already simulated scenarios of risk assessment under reference conditions to realize the conditions imposed by each stress test. For prioritization, a rank measure is introduced for each stress test as a proxy of its priority for simulation-based assessment. The proposed rank measure for stress tests is a function of their potential increase in risks under similar extent of imposed change to their respective input variable. The advantage of ranking stress tests from the highest ratios between input and risks to the least is that it creates a situation of diminishing potential of stress tests in increasing risks. By structuring the stress tests in this way, it is possible to reduce the number of stress tests from an almost infinite number to a few, yielding significant results within a manageable timeframe and with efficient use of computational resources.

It is noted that the proposed methodology does not aim to determine the exact specifications of stress tests that should be conducted to ensure resilience, nor the passing requirements. Rather, it proposes proxies that can be used by decision makers to conduct a rapid screening analysis of candidate stress tests and rank them for further investigation and more detailed simulation-based assessment. This, in turn, saves significant time and effort by narrowing down the long list of potential stress tests to a short list of high-priority ones, those that can lead to overproportionate increase in risks, and allocating resources to conduct only those. The methodology is initially validated through a complex numerical example, and then applied to a real-world case study, which is the road network in the region of Chur, in the canton of Grisons in Switzerland subject to extreme scenarios of flooding.

### Stress testing transport systems

This section reviews existing studies on stress testing the transport systems, a method that can be applied for other critical systems with appropriate adjustments. It is acknowledged that there is extensive literature on risk assessment of transport systems, e.g., [3, 12, 20, 22, 33], to name a few. Nonetheless, only studies focusing on stress testing are reviewed in this paper. Thereafter, the need for prioritizing stress tests and the shortcomings of existing approaches are discussed. Lastly, the mathematical formulation of stress tests, on which the proposed methodology is based, is explained.

As noted earlier, stress tests aim to assess the risks under stressed situations. These stressed situations can be due to various factors, which lead to realization of a new probability distribution for the relevant variables. These factors include the inherent uncertainty associated with input factors, which complicates predictions into the future based solely on past events, particularly the spatiotemporal uncertainty in hydrometeorological events such as flooding [9]. Additionally, external factors can create stressed situations, e.g., climate change exacerbating the intensity of extreme rainfall events or urban development policies driving future increases in travel demand in a road network. These situations, while plausible and probable, are not adequately addressed by current risk assessment methods, hence suggesting the need for stress testing [25].

Several studies have proposed and investigated specific types of stress tests for various systems. In the banking industry for example, stress tests featuring scenarios such as significant increase in the oil price, depreciation of US dollar, and significant increase or decrease in interest rates have been investigated before [36]. In the nuclear industry, stress tests concerning loss of electrical power or malfunctioning of the cooling system have been suggested [16]. Argyroudis et al. [6] investigated stress tests for non-nuclear infrastructure systems.

Despite its demonstrated importance and potential in other domains, research on stress testing within transport systems remains limited. Adey et al. [2] introduced a conceptual framework for developing stress tests, yet it lacks a defined method for conducting them using a simulation-based approach. Nasrazadani et al. [30] proposed a method to conduct simulation-based stress tests for transport systems subject to hydrometeorological events. Based on the proposed method, to conduct simulation-based stress tests, one needs to model the changes that are made to the system under the effect of the stress test, introduce the changes to the system, generate stochastic scenarios, and assess the performance of the system and risks, yet this time under the changes imposed by the stress test.

Other studies focused on defining and conducting specific types of stress tests. Lam et al. [21] investigated asset-based stress tests for road networks using fragility and functional capacity loss functions. Their approach highlights situations where roads and bridges suffer more damage and functionality loss from flooding than expected. Aydin et al. [7] proposed a network theory-based method for conducting asset-based stress tests on road networks exposed to seismic hazards. Their approach involves removing nodes from the network and assessing performance using measures, such as betweenness centrality, thus overlooking network topology and functional aspects, such as travel demand and road capacity. Gauthier et al. [19] integrated stress testing with topological network analysis, presenting asset-based stress tests that evaluate the impact of day-to-day disruptions on travel costs.

Studies on non-asset-based stress tests in transport systems are even scarcer. Nasrazadani et al. [30] investigated three types of such stress tests including increase in the intensity of rainfall events due to climate change, reduction in the number of restoration teams, and increase in the traffic demand. Li et al. [23] also explored the effects of increased traffic load on performance of the network.

These studies, however, are focused only on conducting determined stress tests, without proposing any method on how to select them from a list of candidates, which can considerably reduce the computational effort and incentivize its use. Addressing this gap is the goal of this paper.

### Need for prioritizing stress tests

A significant challenge in analyzing complex infrastructure systems is the large number of potential stress tests that one can or needs to conduct to ensure risks are adequately captured. Apart from specifying the relevant conditions of stress tests, which is extremely challenging, one needs to run simulations for each stress test to evaluate their impact, which can be significantly time consuming. As system complexity grows, so does the computational time needed for accurate testing, potentially extending to several months, e.g., an expected two months of computation time needed for simulation-based assessment of only eight potential scenarios of stress tests for the investigated road network in this article. This highlights the need for a methodology to select the stress tests that, if passed, give sufficient confidence in the system’s resilience.

Although there is no study on prioritizing stress tests, some have proposed the so-called risk-based importance measures. The basic, yet qualitative approach for this purpose is through surveying expert opinions [32]. Several studies utilized quantitative approaches; in particular, network-theory based measures, mainly based on removing a component from the network and assessing its impact on risks [5, 24]. Their approach is, however, limited in measuring the importance of other system variables than those only related to the physical network, e.g., hazard intensity. Other studies proposed importance measures based on SA methods, identifying variables whose variability has a higher contribution to the variability of the system output and hence to the risks. These studies proposed importance measures based on global SA methods, such as Sobol indices [11, 26] and distribution-based SA methods [27]. Nogal and Nogal [34] proposed a novel implementation of SA and importance measures for extreme-based problems, those concerning extreme scenarios of a system, somewhat similar to what is of interest in stress tests. SA studies, however, have two major shortcomings, as explained in the following paragraphs.

The main shortcoming of SA methods, particularly in the context of stress testing, is their inability to capture the impact of changes in the PDF of input variables without additional model evaluation. Global SA techniques determine how much variability in the reference PDF of an input variable leads to variability in the output of the system. Local SA methods typically focus on varying point estimates or deterministic values of input variables, neglecting the broader uncertainty encapsulated by the type, shape, scale, and other characteristics of the underlying PDFs. Stress tests, however, represent a change to the system leading to different PDFs for the relevant variables. Therefore, current implementations of SA and SA-based variable importance measures are limited in the context of stress testing.

Additionally, even if SA methods were extended to account for such changes, their computational burden remains a secondary challenge, particularly for complex systems with several input variables and computationally heavy models. To overcome this issue, studies have proposed solutions such as the use of sensitivity indicators to remove insignificant input variables and reduce the input space [27], and building computationally efficient meta-models, also known as surrogate models, to approximate the system’s complex behavior [13]. Despite improving computational efficiency, meta models have some challenges.

The main challenges of meta models include [4]: (1) the problem of size, which refers to the difficulty in designing simulation experiments and the high computational demand when dealing with a large number of input variables; (2) limitations in applicability due to the calibration of surrogate models using computationally expensive original models and large sample sets; and (3) the need for highly parametrized surrogate models or combinations of surrogate models to achieve adequate accuracy, posing challenges in model selection. In summary, the computational efficiency gained through the surrogate model is offset by a complicated setup demanding technical expertise and a compromise on the precision of the outcomes.

### Mathematical formulation of a stress test

Here, the mathematical formulation to define and conduct simulation-based stress tests per Nasrazadani et al. [30] is explained. It lays the foundation for the proposed methodology, explained subsequently. In the mathematical formulation, ***x*** = {*x*_1_, *x*_2_, …, *x*_*n*_} is the set of *n* input variables representing the system, e.g., intensity of hazard events, characteristics of assets, and number of restoration teams. Most of these variables are uncertain with each having a PDF *x*_*i*_ ~ *f*_*xi*_(*x*), namely, reference PDF. The input variables are correlated with *ρ*_*ij*_ denoting the correlation coefficient between *x*_*i*_ and *x*_*j*_. ***y*** = {*y*_1_, *y*_2_, …, *y*_*m*_} is the set of *m* system outputs of interest, e.g., the costs of repair interventions or the duration of service disruption after a hazard event. The system outputs are a function of the input variables, i.e., ***y*** = *ϒ*(**x**) with function *ϒ*(.) representing the behavior of the system. Depending on the complexity of the system, the function *ϒ*(.) takes various forms, from a closed formulation, as the one discussed in the *Methodology Validation* section, to a complex set of interacting models, as the one discussed in the case study.

The PRA is done through stochastic scenario development using Monte-Carlo simulations based on the PDFs of the input variables, hence generating random realizations of ***x*,** and accordingly ***y***. Given the available computational resources, a set of *N* random scenarios are generated, denoted as ***D*** = {***d***^(1)^, ***d***^(*2*)^, …, ***d***^(*N*)^}, with ***d***^(*j*)^ = {***y***^(*j*)^** | *x***^(*j*)^} = {*y*_1_^(*j*)^, *y*_2_^(*j*)^, …, *y*_*m*_^(*j*)^|* x*_1_^(*j*)^, *x*_2_^(*j*)^, …, *x*_*n*_^(*j*)^} representing the *j*^th^ generated sample point. Using the generated sample points, the empirical PDF of the system outputs can be obtained as *y*_*i*_ ~ *f*_*yi*_(*y*).

To evaluate risks, a set of risk measures is defined based on the system outputs. ***R*** = {*R*_1_, *R*_2_, …, *R*_*r*_} represents the set of *r* risk measures, defined as a function of the system outputs, *R*_*i*_ = *Ω*_*i*_(***y***). The function *Ω*(.) calculates a statistic from the PDF of relevant outputs, e.g., mean or higher moments, or a certain percentile. For example, a risk measure could be the average duration of service recovery or the 95 th percentile of costs of restoration interventions after a hazard event. Risks are then assessed with respect to pre-defined acceptable thresholds.

Upon completion of the reference risk assessment, stress tests can be defined and conducted. Each stress test is represented by a set of conditions, which are imposed on the system to achieve the new system representation under the effect of the stress test. The same scenario generation is then done, yet this time with the relevant conditions of the stress test in place, leading to new realizations of the system outputs, hence new values of the risk measures. New or similar to the original acceptable thresholds are then used to assess whether risks under the effect of each stress test are acceptable or not, indicating pass/failure of the stress test.

In the mathematical formulation, *π*_*s*_ represents a stress test from the set of *S* candidate stress tests ***π*** = {*π*_1_, *π*_2_, …., *π*_*S*_}. *x*|*π*_*s*_ and *f*_*x*_|*π*_*s*_(*x*) represent system variables and their PDFs under the effect of the stress test *π*_*s*_, respectively. Next, *N*|*π*_*s*_ random sample points of the system are generated again and the system outputs are recalculated as ***y***|*π*_*s*_ = *ϒ*(***x***|*π*_*s*_) with their new PDFs as *y*_*j*_ ~ *f*_*yj*|_*π*_*s*_(*y*). Lastly, the risk measures under the effect of the stress test, ***R***|*π*_*s*_ = *Ω*(***y***|*π*_*s*_), are evaluated to assess pass/failure of the stress test using acceptable thresholds. Risk reducing interventions affecting variables whose relevant stress tests fail can be then further studied.

The general stress testing framework allows for conducting stress tests that feature multiple variables, and hence the generic notation *π*_*s*_ is initially employed. However, the prioritization methodology proposed in this study exclusively targets single-variable stress tests, and therefore, in the rest of this paper, the notation *π*_*i*_ is used, implying that stress test is corresponding to variable *x*_*i*_.

It should be noted that the notation *x*|*π*_*s*_ and *f*_*x*_|*π*_*s*_(*x*) employed herein represents a generalized concept distinct from classical conditional probability. While classical conditional probability involves conditioning on known realizations of system variables, the concept adopted in the context of stress testing is broader. It includes not only classical conditioning scenarios but also hypothetical alterations to underlying assumptions or distributions of system variables.

### Methodology to prioritize stress tests

**Fig. 1:**
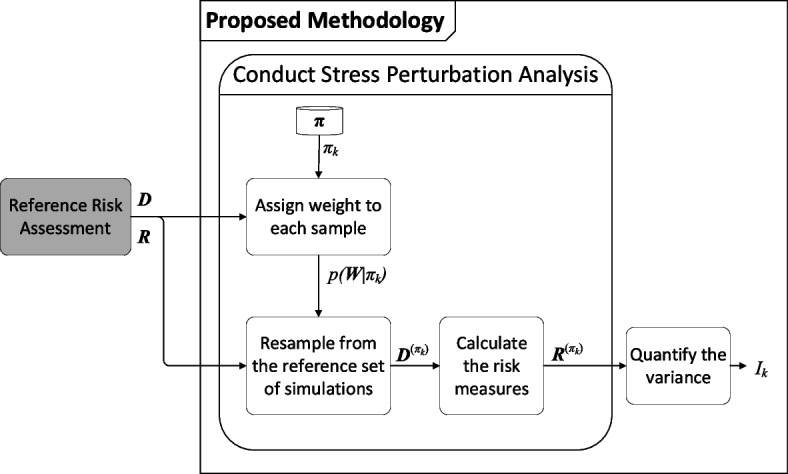
Schematic workflow of the proposed methodology; Inputs are generated reference sample points (***D***) and references values of risk measures (***R***), and outputs for an example stress test *π*_*k*_ are resampled sample points (***D***^(*π*^_*k*_^)^), updated values of risk measures (***R***^(*π*^_*k*_.^)^), and rank measure of the stress test (*I*_*k*_)

In this section, the proposed methodology to prioritize stress tests is presented. In essence, the methodology utilizes the results of an existing reference risk assessment (represented by previously simulated sample points and their associated risk measures) and estimates how hypothetical stress-test scenarios would impact these risks without the need for additional computationally intensive simulations. As schematically shown in Fig. 1, it includes five modules, which are explained in detail in the following subsections. First, the method assigns importance weights to each existing simulation sample, reflecting their likelihood under a given stress-test scenario. These weights are calculated based on a novel implementation of importance sampling principles. Next, using these weights, the method employs a bootstrap resampling approach to generate new datasets that represent scenarios under stressed conditions. The risk measures are recalculated using these resampled datasets, allowing estimation of scenario impacts without further simulation runs.

To systematically evaluate stress tests, the methodology includes a stress perturbation analysis, where the imposed stress levels are incrementally increased (or decreased) for each stress test, enabling a systematic examination of how varying stress intensities impact risks. Subsequently, a rank measure indicating the priority of each stress test for detailed simulation-based analysis is introduced. This measure serves as a practical proxy to highlight which stress scenarios are most likely to cause disproportionate increases in risk and therefore should be prioritized for further, detailed evaluations.

### Assign weight to each sample point

Consider a stress test denoted as *π*_*i*_, where *x*_*i*_ as one of the input variables is subjected to stress, leading to a modification in its PDF. For each sample point ***d***^(*j*)^ = {***y***^(*j*)^** | *x***^(*j*)^} of the reference assessment, a weight factor, denoted as *w*_*i*_^(*j*)^, is calculated as the ratio of the new PDF of *x*_*i*_ to its reference, as follows:

1 $$w_{i}^{\left( j \right)} = \frac{{\left. {f_{{x_{i} }} } \right|\pi_{i} \left( {x_{i}^{\left( j \right)} } \right)}}{{f_{{x_{i} }} \left( {x_{i}^{\left( j \right)} } \right)}}, \, j = 1,2, \ldots ,N$$

A discrete random variable with as many outcomes as the number of sample points *N*, denoted as **W**_*i*_ = {*w*_*i*_^(1)^_,_ *w*_*i*_^(2)^_, …,_ *w*_*i*_^(*N*)^}, is accordingly defined as:

2 $$p\left( {{\mathbf{W}}_{i} = \left. {w_{i}^{{^{\left( j \right)} }} } \right|\pi_{i} } \right) = \left. {p_{j} } \right|\pi_{i} = \frac{{w_{i}^{{^{\left( j \right)} }} }}{{\Sigma_{j = 1}^{N} w_{i}^{{^{\left( j \right)} }} }}$$

This ratio is analogous to the *likelihood ratio* in importance sampling method [39], which shows the ratio between the target PDF to the one that is sampled from. The weight factor shows to which extent each generated sample point under reference conditions, ***d***^(*j*)^, is likely to occur under stressed conditions. It can be implied that if the system remained in the reference conditions, i.e., *w*_*i*_^(*j*)^ = 1, **W**_*i*_ would have a uniform PDF with *p*_*j*_ = 1/*N*. For a stress test, however, it can be inferred that those sample points with *p*_*j*_*|π*_*i*_ > 1/*N* are more likely to occur under the effect of the considered stress test and vice versa. In other words, under reference conditions, *p*(***D*** = ***d***^(*j*)^) = 1/*N*, known as the prior probability, and under the stress test *π*_*i*_, *p*(***D*** = ***d***^(*j*)^|*π*_*i*_) = *p*_*j*_*|π*_*i*_, i.e., the posterior probability. As an example, an extreme rainfall event is more likely to happen under climate change stress tests compared to the likelihood of the same event under reference assessment.

It is noted that *p*_*j*_ does not indicate how likely it is for a sample point to be realized. Rather, it shows the relative contribution of each sample point, among the generated sample, to the estimation of the outcomes of the system, e.g., risk. Therefore, all sample points are treated equally under reference conditions, i.e., ∀*j* ∈ {1, 2,…, *N*}; *p*(***D*** = ***d***^(*j*)^) = 1/*N*. Yet, under stressed situations, sample points generated under reference conditions would contribute differently to estimating the risks.

### Resample from the reference set of simulations

In this step, a Bootstrapping technique is used to generate a resampled set of the reference set of simulations, namely, a bootstrap sample. To generate a bootstrap sample, given the PDF of **W**_*i*_*|π*_*i*_, a random instance is drawn from **W**_*i*_*|π*_*i*_, e.g., *w*_*i*_^(j*)^, which in turn determines that sample point ***d***^(j*)^ = {***y***^(j*)^** | *x***^(j*)^} is included in the resampled set. This random selection can be repeated multiple times, i.e., as many times as *N*|*π*_*i*_, to obtain the resampled set ***D***^(*π*^_*i*_^)^ = Д(***D***, *p*(**W**_*i*_*|π*_*i*_)), where Д(.) is the resampling function. Referring to Fig. 1, this module receives the PDF of the weights of reference sample points and generates a bootstrap sample ***D***^(*π*^_*i*_^)^.

Suppose that *f*_*xi*_^(*π*^_*i*_^)^(*x*) represents the PDF of the instances of *x*_*i*_ in the resampled dataset. According to the principles of importance sampling method [39], considering that weights are defined based on the ratio between *f*_*xi*_|*π*_*i*_(*x*), as the target PDF, to *f*_*xi*_(*x*), as the PDF where sample points are selected from, it can be inferred that *f*_*xi*_^(*π*^_*i*_^)^(*x*) is a statistical estimator for *f*_*xi*_|*π*_*i*_(*x*), denoted as *f*_*xi*_^(*π*^_*i*_^)^(*x*) ≈ *f*_*xi*_|*π*_*i*_(*x*). Therefore, selected instances of *x*_*i*_ in the resampled dataset follow the same PDF that is dictated by the stress test *π*_*i*_. It will be later discussed in the *Discussion* section what factors affect the quality of this estimator. For variables other than *x*_*i*_, if *x*_*k*_ is independent from *x*_*i*_, resampling based on *x*_*i*_ would not have any impact on its PDF, i.e., *f*_*xk*_^(*π*^_*i*_^)^(*x*) ≈ *f*_*xk*_(*x*). However, if the two variables are correlated, change in the PDF of one would have an impact on the PDF of the other.

Since resampling is solely based on the PDF of *x*_*i*_ and sample points are selected from the reference dataset, the correlation coefficient between the resampled values of any two variables remains unchanged, i.e., *ρ*_*jk*_^(*π*^_*i*_^)^ = *ρ*_*jk*_ for any *j* and *k*. In other words, the proposed methodology assumes that the correlation structure of the system variables is preserved under stress tests. For example, when formulating a stress test targeting at increased rainfall intensity, the proposed resampling approach effectively captures its consequential impact on flood event intensity, given their correlation. It is acknowledged, however, that preservation of the correlation structure in the proposed methodology is an assumption and the extent to which it is applicable to any particular situation can be different and lies at the discretion of the decision maker. If it is assumed that a stress test alters the correlation structure of the system, the proposed methodology will need to be adapted, which is the topic of future research.

### Calculate the risk measures

The resampled set ***D***^(*π*^_*i*_^)^ replicates scenarios that would be generated if the stress test were simulated. This means that the PDFs of the system outputs in the resampled dataset, *f*_*y*_^(*π*^_*i*_^)^(*y*), are also an estimator of the PDFs of the system outputs if the stress test were simulated, *f*_*y*_|*π*_*i*_(*y*). This leads to the conclusion that the values of the risk measures calculated using the resampled dataset, denoted as ***R***^(*π*^_*i*_^)^, are estimators for the risk measures based on simulations, denoted as ***R***|*π*_*i*_. Therefore, without conducting new simulations, and only based on a computationally free resampling approach, the impacts of stress tests on risks can be estimated.

It is noted that the bootstrap resampling in the proposed methodology can be done multiple times. Therefore, for each stress test, multiple bootstrap samples can be generated leading to having multiple estimations of the value of the risk measures. This has a twofold benefit. First, it can provide confidence intervals for the values of risk measures. Second, it allows assessing how robust the estimations of the risk measures are, given different sets of reference simulations as the input, discussed later. This is, in fact, one of the uses of bootstrap resampling, which is to assess the variability and robustness of a statistical model or estimator [38].

### Conduct stress perturbation analysis

To gain more insight into how the imposed stresses impact risks, e.g., to understand which levels of imposed stresses would lead to having risks exceeding the acceptable threshold, a stress perturbation analysis is conducted. For that, the imposed stress by the relevant stressor is increased in a stepwise manner up to the desired level. For example, for a stress test that considers the increase in the intensity of rainfall events, one can consider 10 stress levels with 1% increments, leading to 10 scenarios of variable increases, resulting in 10 PDFs for the variable. Similarly, multiples levels of relieving stress can be evaluated. This, as will be shown later, can help identify the dominant variables in the risk reduction. Since the proposed resampling approach is computation-free, the values of the risk measures under the effect of each of these scenarios can be readily estimated.

**Fig. 2:**
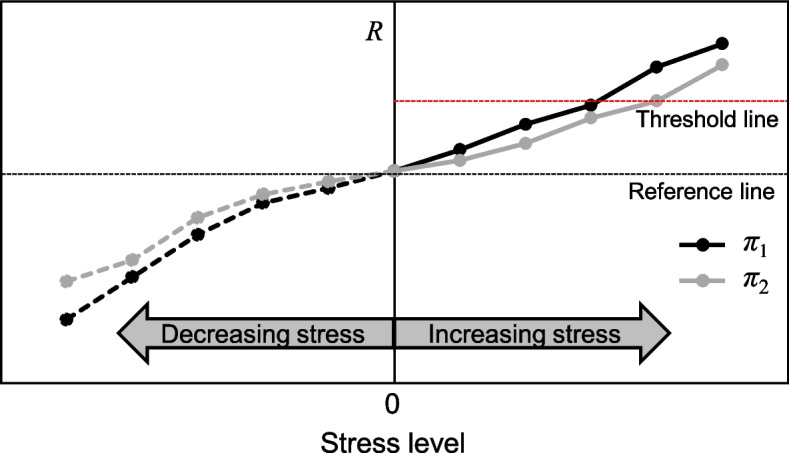
Example results of stress perturbation analysis of stress tests *π*_1_ and *π*_2_ when increasing or decreasing stress with respect to the initial conditions (i.e., reference values)

Figure 2 shows an example output of the stress perturbation analysis for two stress tests *π*_1_ and *π*_2_, where the vertical axis is a risk measure, *R,* and horizontal axis is the stress level, quantified in the next subsection. The reference line represents the results of the reference assessment, where stress level is essentially zero. The threshold line shows the maximum acceptable risk. Referring to the right half of the figure, it can be seen that increase in the level of stress in stress test *π*_1_ is expected to result in higher increase in *R*. Additionally, an estimation of the stress level at which *R* exceeds the threshold line can be obtained for each stress test.

The proposed methodology also allows for analyzing hypothetical stress reduction scenarios, implying improvement in the system by the aid of some interventions. This is captured by the curves in the left side of the Fig. 2. This analysis identifies which variables, if improved through targeted interventions, could yield higher risk reductions, and to what extent.

### Quantify the variance

The proposed methodology ranks stress tests based on the variance of the risk measures with respect to the reference condition, adjusted based on the level of stress. The level of stress is represented by the difference between the stressed and reference PDFs of the relevant variable. Measuring the difference, or discrepancy, between two PDFs, has already been treated in literature by the Kolmogorov–Smirnov statistic [10], where the largest absolute distance between two cumulative distribution functions (CDF) is the indicator of how different the corresponding PDFs are. This enables a consistent treatment of various stress tests, e.g., one that concerns an increase in the mean value of a variable, one that is about increasing the standard deviation, and one that is about a change in the shape of the PDF. The proposed rank measure is dimensionless, defined between [0,1], and is calculated as follows:

3 $$I_{i,k}^{s} = \frac{{\sum\limits_{v = 1}^{{v = V_{i} }} {\frac{1}{{V_{i} }}\left( {\frac{{R_{k}^{{\left( {\pi_{{_{{x_{i} }} }}^{{\left\{ v \right\}}} } \right)}} - R_{k} }}{{\xi \left| {\pi_{{x_{i} }}^{{\left\{ v \right\}}} } \right.}}} \right)}^{2} }}{{\sum\limits_{i = 1}^{n} {\sum\limits_{v = 1}^{{v = V_{i} }} {\frac{1}{{V_{i} }}\left( {\frac{{R_{k}^{{\left( {\pi_{{_{{x_{i} }} }}^{{\left\{ v \right\}}} } \right)}} - R_{k} }}{{\xi \left| {\pi_{{x_{i} }}^{{\left\{ v \right\}}} } \right.}}} \right)}^{2} } }}$$

where *I*^*s*^_*i*_*,*_*k*_ is the rank measure indicating the priority of the stress test that can be defined based on *x*_*i*_ with respect to risk measure *R*_*k*_, *π*_*xi*_^{*v*}^ is the stress test defined based on variable *x*_*i*_ with the stress level of *v*, *V*_*i*_ is the number of stress levels, *R*_*k*_^(π^_*xi*_^)^ is the estimation of risk measure based on the results of the resampling, and *ξ*|*π*_*xi*_^{*v*}^ is the Kolmogorov–Smirnov statistic, comparing the stressed PDF and reference PDF of *x*_*i*_, calculated as follows. Note that the Kolmogorov–Smirnov statistic *ξ*|*π*_*xi*_^{*v*}^ explicitly depends on the stress level *v*, as each stress level defines a different CDF for the corresponding variable.

4 $$\xi \left| {\pi_{{x_{i} }}^{{\left\{ v \right\}}} } \right. = \mathop {\max }\limits_{{x_{i} }} \left| {\left. {F_{{x_{i} }} } \right|\pi_{{x_{i} }}^{{\left\{ v \right\}}} \left( x \right) - F_{{x_{i} }} \left( x \right)} \right|$$

The aggregate rank measure for each stress test, i.e., one considering all the risk measures, denoted as *I*_*i*_, can be calculated as the average or weighted average of the individual rank measures corresponding to risk measures.

Note that in Eq. (3), the change in the risk measure is normalized by the stress level. This is due to the fact that imposing more stress on each variable results in a higher increase in risk and accordingly higher variance. This can misleadingly affect the rank of the variable, i.e., variables that are subjected to higher stress levels might misleadingly show themselves to have a higher priority. To avoid this, the change in the risk measure is adjusted by the stress level. By the same token, and to maintain consistency, the calculation of rank measure for each stress test is done using the same range of stress level for all stress tests. For example, if the stress level range for two stress tests are [0 – *ξ*|*π*_*xi*_^{*Vi*}^] and [0 – *ξ*|*π*_*xj*_^{*Vj*}^], where *ξ*|*π*_*xi*_^{*Vi*}^ < *ξ*|*π*_*xj*_^{*Vj*}^, the calculation of the proposed measures for both stress tests is done up to the lowest level. This would avoid drawing conclusions based on consideration of stress levels that have not been tested for some stress tests.

The proposed measure can also be calculated under situations where variables undergo stress reduction. This allows identifying variables that can have a more significant impact on reducing the risks. Following a similar approach as given by Eq. (3), yet by reversing the imposed stress level, the rank measure of variables when subjected to stress reduction can be quantified. To make distinction, this rank measure is denoted as *I*^*p*^_*i*_*,*_*k*_*.*

### Methodology validation

To validate the proposed methodology, this section shows its performance when compared to running simulations on a complex hypothetical system previously used by various researchers to demonstrate the performance of their methods. This example is based on the one discussed in multiple studies on SA [14, 34, 41]. It features a mathematical equation using two input variables ***x*** = {*x*_1_, *x*_2_}. It can be assumed that this equation represents the behavior of a complex hypothetical system with *y* = *ϒ*(***x***) being its single output of interest, defined as:

5 $$\begin{gathered} y = \Upsilon \left( {x_{1} ,x_{2} } \right) = 0.2\exp \left( {x_{1} - 3} \right) + 2.2\left| {x_{2} } \right| + 1.3x_{2}^{6} - 2x_{2}^{2} - 0.5x_{1}^{4} + 2.5x_{1}^{2} \hfill \\ \, + 0.7x_{1}^{3} + \frac{3}{{\left( {8x_{1} - 2} \right)^{2} + \left( {5x_{2} - 3} \right)^{2} + 1}} + \sin \left( {5x_{1} } \right)\cos \left( {3x_{1}^{2} } \right) \hfill \\ \end{gathered}$$

Input variable *x*_1_ follows a normal PDF with zero mean and 0.3 as its standard deviation, i.e., *x*_1_ ~ *N*(*μ*_1_ = 0, *σ*_1_^2^ = 0.3^2^). Input variable *x*_2_ follows a generalized beta distribution with parameters *α* = 2 and *β* = 2 defined on the interval (‒1, 1), i.e., *x*_2_ ~ *G-Beta*(*α* = 2, *β* = 2, a = ‒1, *b* = 1). The mean and standard deviation can also be calculated based on these four parameters as *μ*_2_ = 0, *σ*_2_ = 0.447. It is noted that *x*_1_ follows an unbounded PDF, while the PDF of *x*_2_ is bounded. Additionally, it is assumed that the two variables are correlated with a correlation coefficient of *ρ*_1,2_ = 0.25. For illustrative purposes, three risk measures are considered, the mean, median, and 95 th percentile of the system output *y*, i.e., *R*_1_ = *ȳ*, *R*_2_ = *y*_50p_, and *R*_3_ = *y*_95p_.

For illustrative purposes, two stress tests are considered, one featuring an increase in the values of the variable *x*_1_, namely *π*_*x*1_, and one featuring an increase in the likelihood of higher values in the distribution of the variable *x*_2_, namely *π*_*x*2_. Both stress tests, as shown later, result in an increase in risks.

In the following, first the results of the reference risk assessment are presented. Next, the results of the stress test assessment for an example scenario of the stress test *π*_*x*2_ are shown. Then, the results of the stress perturbation analysis for both stress tests are shown and the rank measures are quantified. Lastly, the results of the proposed methodology are validated with the results obtained from evaluating stress tests using simulations.

### Reference assessment

**Fig. 3:**
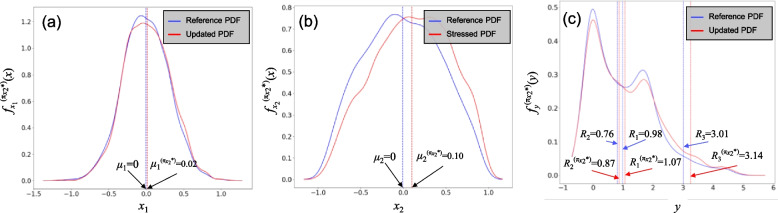
PDFs of input variables and system outputs under reference condition and under *π*_*x*2_^*^: a) *x*_1_, b) *x*_2_, and c) *y*

For reference risk assessment, *N* = 5,000 random sample points were generated. Figure 3 show the results of the reference risk assessment, together with the example stress test, explained next. In particular, Fig. 3a-c shows the PDFs of variables *x*_1_ and *x*_2_, and system output *y*, respectively. The blue lines show the reference PDFs, which are based on the generated sample points of input variables following their defined PDF. The red lines show the PDFs of the variable directly stressed *f*_*x*2_^(*π*^_*x*2_^*)^(*x*), and the updated PDFs of other variables not directly stressed, *f*_*x*1_^(*π*^_*x*2_^*)^(*x*), and outputs, *f*_*y*_^(*π*^_*x*2_^*)^(*x*), explained next. Given the PDF of the system output, the considered risk measures under reference condition are obtained as *R*_1_ = 0.98, *R*_2_ = 0.76, and *R*_3_ = 3.01, also shown on Fig. 3c.

### Candidate stress tests

In this study, two stress tests are considered: *π*_*x*1_ features a shift in the entire PDF of *x*_1_, and *π*_*x*2_ features an increase in the likelihood of above-mean values of *x*_2_, while maintaining its bounds and variance. Both stress tests can be modelled by changing the mean value of the relevant variable and keeping other parameters fixed. An example scenario for these two stress tests could be a 0.1 increase in their mean value. Table 1 shows the two example stress tests and the imposed conditions on the PDF of their variable.

**Table 1:** Example stress test scenarios in the numerical example

Variable	*x*_1_	*x*_2_
Reference PDF	*x*_1_ ~ *N*(*μ*_1_ = 0, *σ*_1_^2^ = 0.3^2^)	*x*_2_ ~ *G-Beta*(*α* = 2, *β* = 2, a = ‒1, *b* = 1)
Stress test	*π*_*x*1_^*^ = 0.1 increase in the mean value	*π*_*x*2_^*^ = 0.1 increase in the mean value, though maintaining the boundaries and variance
Imposed conditions on corresponding variable	$$\begin{gathered} \left. {\mu_{1} } \right|\pi_{{x_{1} }}^{*} = \mu_{1} + \left( {0.1} \right) \, = \, 0.1 \hfill \\ \left. {\sigma_{1} } \right| \pi_{{x_{1} }}^{*} = \sigma_{1} = { 0}{\text{.3}} \hfill \\ \end{gathered}$$	$$\left\{ {\begin{array}{*{20}l} {\left. {\mu_{2} } \right|\pi_{{x_{2} }}^{*} = \mu_{2} + \left( {0.1} \right) \, = \, 0.1} \hfill \\ \begin{gathered} \left. {\sigma_{2} } \right| \pi_{{x_{2} }}^{*} = \sigma_{2} = \, 0.447 \hfill \\ \left. a \right|\pi_{{x_{2} }}^{*} = a = - 1 \hfill \\ \end{gathered} \hfill \\ {\left. b \right|\pi_{{x_{2} }}^{*} = b = \, 1} \hfill \\ \end{array} } \right. \Rightarrow \left\{ \begin{gathered} \left. {\alpha_{2} } \right|\pi_{{x_{2} }}^{*} = 2.17 \hfill \\ \left. {\beta_{2} } \right| \pi_{{x_{2} }}^{*} = 1.78 \hfill \\ \end{gathered} \right.$$
Stressed PDF	*x*_1_ ~ *N*(*μ*_1_*|π*_*x*1_^*^ = 0.1, *σ*_1_*|π*_*x*1_^*^ = 0.3^2^)	*x*_2_|*π*_*x*2_ ~ *G-Beta*(*α|π*_*x*2_^*^ = 2.17, *β|π*_*x*2_^*^ = 1.78, *a|π*_*x*2_^*^ = ‒1, *b|π*_*x*2_^*^ = 1)

The first step is to calculate the weights for each of the 5,000 generated reference sample points under each stress test. For brevity, however, the detailed explanation of the resampling approach is provided only for stress test *π*_*x*2_^*^ in this section. The same resampling approach has been applied for *π*_*x*1_^*^. Given the *G-Beta* PDF, and the distribution parameters under reference and stress test conditions as given per Table 1, these weights can be calculated as follows:

6 $$w_{2}^{\left( j \right)} = \frac{{\left. {f_{{x_{2} }} } \right|\pi_{{x_{2} }} \left( {x_{2}^{\left( j \right)} } \right)}}{{f_{{x_{2} }} \left( {x_{2}^{\left( j \right)} } \right)}} = 2^{0.05} \left( {x_{2}^{\left( j \right)} + 1} \right)^{0.17} \left( {1 - x_{2}^{\left( j \right)} } \right)^{ - 0.22} \frac{{\int_{0}^{{x_{2}^{\left( j \right)} }} {t^{1.17} } \left( {1 - t} \right)^{0.78} dt}}{{\int_{0}^{{x_{2}^{\left( j \right)} }} t \left( {1 - t} \right){\mkern 1mu} dt}}, \, j = 1,2, \ldots ,N = 5000$$

Subsequently, the discrete PDF of the **W** is obtained, based on which the resampling can then be done using the 5000 reference simulations. Figure 3a and b show, in red color, the updated PDF of *x*_1_ and the stressed PDF *x*_2_, respectively. These new PDFs are based on the resampled sample points from the reference sample. It can be seen that the resampling approach could capture the condition imposed by the stress test, which is the 0.1 increase in the mean value of *x*_2_. Lastly, Fig. 3c shows the updated PDF of the system output *y*. Accordingly, the considered risk measures are estimated as *R*_1_^(π^_*x*2_^*)^ = 0.87, *R*_2_^(π^_*x*2_^*)^ = 1.07, and *R*_3_^(π^_*x*2_^*)^ = 3.14.

### Prioritizing stress tests

The next step is to conduct the stress perturbation analysis. For the considered stress tests, the extent of change to the mean is essentially the lever to change the stress level. For illustrative purposes, the range from 0 to 0.2 increase in the mean with 0.05 increments is considered for both stress tests. For the analysis of stress reduction, in this case decreasing the mean of the variables, the same range is considered.

**Fig. 4:**
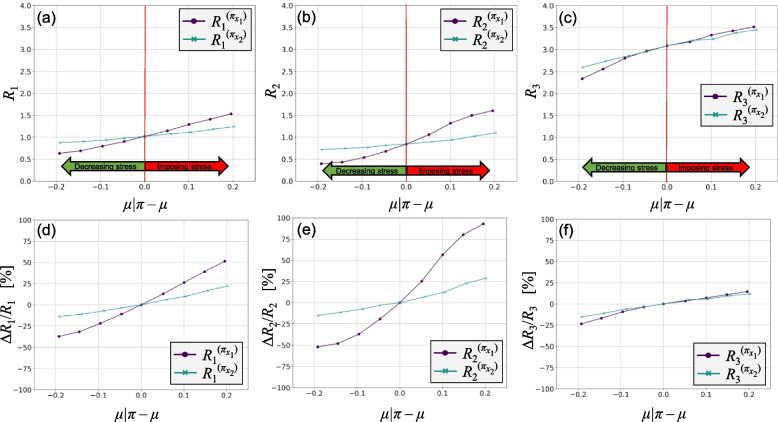
Stress perturbation analysis for the numerical example

Figure 4 shows the results of the stress test perturbation analysis. Figure 4a-c shows the estimated values of the risk measures with respect to the change in the mean value of the relevant variable, i.e., *μ*|*π* ‒ *μ*. As shown, positive values of change to the mean result in increasing all risk measures. Figure 4d-f show the relative change in the estimated values of the risk measures. For risk measures *R*_1_ and *R*_2_, stress test *π*_*x*1_ shows a more significant impact on risks than *π*_*x*2_. However, for risk measure *R*_3_, it seems that both stress tests have a relatively similar impact under similar change to the mean of their respective variable.

**Fig. 5:**
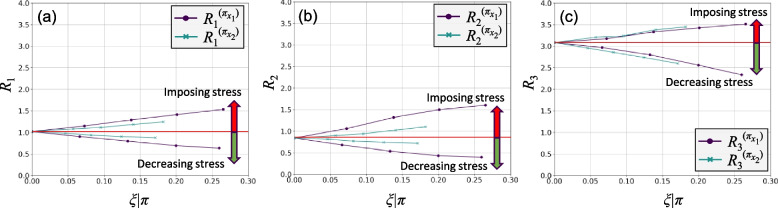
Risk measures vs the Kolmogorov–Smirnov statistic (*ξ*|*π*)

Each level of change to the mean of each variable corresponds with a Kolmogorov–Smirnov statistic as a representation of the level of change to its PDF. Figure 5 shows the estimated values of the risk measures against the Kolmogorov–Smirnov statistic for each stress test, *ξ*|*π*. The upper part of each figure, as delineated by the red line, shows imposing stress scenarios and increase in risks, as opposed to the lower part, which represents stress reduction. It can be seen that stress test *π*_*x*1_ results in greater stress level under similar extent of change to the mean of its respective variable. For risk measures *R*_1_ and *R*_2_, stress test *π*_*x*1_ shows a greater impact on risks, while for risk measure *R*_3_, *π*_*x*2_ has a slightly greater impact. This is also reflected in their rank measures, explained shortly.

Table 2 shows the calculated rank measures for each stress test with respect to the risk measures for both scenarios of imposing stress and reducing. Stress test *π*_*x*1_ has a higher rank than *π*_*x*2_ with respect to *R*_1_ and *R*_2_, while for *R*_3_, *π*_*x*2_ has a higher rank. This suggests that imposing stress on variable *x*_1_ is more likely to result in higher increase in *R*_1_ and *R*_2_, and less in *R*_3_. To obtain the aggregate rank measure, the average of individual rank measures is considered for this example. Stress test *π*_*x*1_ was shown to have a higher aggregate rank measure. This suggests that to explicitly conduct the stress tests using simulations and thus to allocate time and resources for this matter, *π*_*x*1_ has higher priority to be included as a stress test.

**Table 2:** Rank measures for *π*_*x*1_ and *π*_*x*2_ in the numerical example

	Imposing stress				Decreasing stress			
Stress test	*R*_1_	*R*_2_	*R*_3_	Aggregate	*R*_1_	*R*_2_	*R*_3_	Aggregate
*π*_*x*1_	*I*^*s*^_1_*,*_1_ = 0.75	*I*^*s*^_1,2_ = 0.89	*I*^*s*^_1,3_ = 0.43	*I*^*s*^_1_ = 0.69	*I*^*p*^_1,1_ = 0.75	*I*^*p*^_1,2_ = 0.82	*I*^*p*^_1,3_ = 0.43	*I*^*p*^_1_ = 0.67
*π*_*x*2_	*I*^*s*^_2,1_ = 0.25	*I*^*s*^_2,2_ = 0.11	*I*^*s*^_2,3_ = 0.57	*I*^*s*^_2_ = 0.31	*I*^*p*^_2,1_ = 0.25	*I*^*p*^_2,2_ = 0.18	*I*^*p*^_2,3_ = 0.57	*I*^*p*^_2_ = 0.33

### Validation

**Fig. 6:**
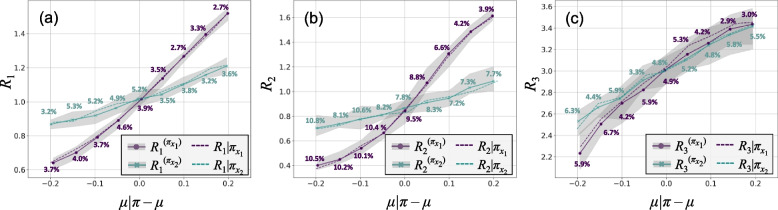
Comparison of the results obtained through simulations, *R*|*π*, and by applying the proposed methodology, *R*^(*π*)^, with different resampled sets for a) *R*_1_, b) *R*_2_, and c) *R*_3_

In this section, the obtained results are validated with respect to the results of evaluating stress tests using simulations. Additionally, the robustness of the proposed methodology with respect to the input samples is assessed. Figure 6 shows the results of the validation analysis. For each stress test scenario, resampling was repeated 20 times, providing 20 estimations for each risk measure, *R*^(*π*)^, and hence a potential range for such estimations. The range of estimations is shown by grey areas, with solid lines representing the average results of these estimations. The dashed lines show the results of stress tests using simulations, *R*|*π*. As seen, the simulation results closely align with the average estimation results obtained from the proposed methodology.

The percentage values in each figure show the maximum relative difference between *R*|*π* and *R*^(*π*)^. Note that results from simulating stress tests also have variation, as generating new reference samples can lead to new risks. Therefore, variation of results for the reference situation, i.e., *μ*|*π* = *μ*, essentially sets the baseline to check whether the variability of results for different stress levels is consistent with that of the reference situation, hence suggesting the robustness of the method. Referring to Fig. 6, it can be seen that the range of variation over stress test scenarios is consistent with that of the reference situation, hence indicating that the proposed methodology is robust with respect to the input sets of simulations.

The reference sample serves as the basis for estimating risks under stressed conditions, and its representativeness directly impacts the accuracy of the estimation. If the reference sample fails to capture the full range of possible scenarios or does not adequately reflect the system’s complexities, the accuracy of the resulting risk estimates based on resampling may be compromised. That is, larger deviation of risk estimations, using the proposed methodology, from the results of simulations is expected.

**Fig. 7:**
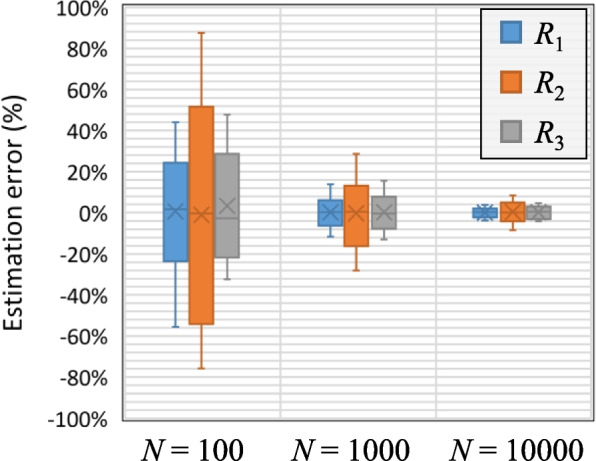
Estimation error of the risk measures with respect to the number of reference simulations

Figure 7 shows the range of estimation error for the three considered risk measures for *N* = 100, *N* = 1′000, and *N* = 10′000. The estimation error is the relative difference between the estimated risk measure obtained from resampling to the mean values of 10 evaluations of the risk measure, each based on conducting *N* simulations. It is evident the range of error for all risk measures is improved as the number of input simulations increases. Nevertheless, it is noteworthy to observe that across all risk measures and for various cases of the number of input simulations, the mean estimation error consistently remains remarkably close to zero deviation. This highlights the importance of repeating the resampling approach to achieve more accurate results, and the capability of the proposed methodology in facilitating this without inducing any computational burden.

To further explore the impact of sample size on the accuracy of the proposed methodology, a complementary analysis was conducted and summarized in Table 3. This analysis considers both simulation-based and estimation-based evaluations of risk measures across the same three sample sizes (100, 1,000, and 10,000) and for a range of changes to the variables. For each case, simulations were conducted with 10 independent resampling iterations to characterize the variability in calculated risk measures, while the proposed method was also applied with 10 resampling iterations to estimate the same measures. The average risk values obtained from the simulations with 10,000 samples were used as a benchmark for evaluating accuracy.

**Table 3:** Effect of sample size on risk estimation accuracy: simulation vs. proposed method

Change in variable	Risk measure	*N* = 100		*N* = 1000		*N* = 10,000	
		Simulation variability ^a^	Estimation variability ^b^	Simulation variability ^a^	Estimation variability ^b^	Simulation variability ^a^	Estimation variability ^b^
‒ 0.1	*R*_1_	1.9% [‒23, + 45]%	2.1% [‒26, + 44]%	0.40% [‒8, + 13]%	0.42% [‒10, + 14]%	0% [‒3, + 3]%	0.01% [‒3, + 3]%
	*R*_2_	3.9% [‒60, + 66]%	4.0% [‒62, + 64]%	1.7% [‒17, + 28]%	1.8% [‒19, + 29]%	0% [‒7, + 8]%	0.03% [‒7, + 8]%
	*R*_3_	2.8% [‒32, + 26]%	3.0% [‒30, + 30]%	0.80% [‒11, + 10]%	0.83% [‒12, + 10]%	0% [‒3, + 3]%	0.02% [‒4, + 3]%
0.0	*R*_1_	1.6% [‒27, + 39] %	N.A	0.12% [‒5, + 6]%	N.A	0% [‒1, + 2]%	N.A
	*R*_2_	3.1% [‒70, + 77]%	N.A	0.19% [‒15, + 13]%	N.A	0% [‒8, + 5]%	N.A
	*R*_3_	2.0% [‒28, + 31]%	N.A	0.13% [‒5, + 12]%	N.A	0% [‒3, + 3]%	N.A
+ 0.1	*R*_1_	1.9% [‒14, + 16]%	1.8% [‒17, + 17]%	0.33% [‒7, + 6]%	0.31% [‒7, + 6]%	0% [‒1, + 1]%	0.00% [‒1, + 2]%
	*R*_2_	2.7% [‒31, + 25]%	2.9% [‒33, + 23]%	0.84% [‒11, + 20]%	0.89% [‒12, + 21]%	0% [‒3, + 4]%	0.02% [‒3, + 4]%
	*R*_3_	2.2% [‒18, + 46]%	2.3% [‒21, + 47]%	0.51% [‒9, + 8]%	0.53% [‒9, + 9]%	0% [‒4, + 3]%	0.02% [‒4, + 3]%

^a^Variability of the values of risk measures using simulations

^b^Variability of the estimated values of risk measures using the proposed method

The results show that sample size influences the accuracy of both simulation outputs and our method’s estimations. Importantly, the variability observed in the estimation closely aligns with that from simulations, confirming that the proposed method remains robust under different stress levels and sampling conditions. Moreover, the analysis reinforces that repeating the resampling process—even a modest number of times—can significantly improve estimation accuracy. For example, under a ‒0.1 stress applied to the variables and risk measure *R*_1_, the average estimation error was only 2.1%, compared to 1.9% in the simulation-based results. These findings demonstrate that repeated resampling is a practical and effective approach to augment limited sample sizes, enhancing the accuracy of risk estimation without the need for additional simulations.

The validation analysis conducted in this section was an essential step before demonstrating the methodology’s effectiveness in a real-world case study, as shown subsequently. To achieve this, the method was validated using a hypothetical system defined by a complex mathematical formulation—an approach commonly used in the literature for evaluating methods designed to analyze the impacts of changes on complex systems, including sensitivity analyses. This step was necessary to confirm that the proposed method can reliably capture various intricate behaviors expected in real-world applications, thereby ensuring robustness and general applicability beyond any specific scenario or context. Performing this validation thus provided confidence that the methodology is capable of identifying impactful stress-test scenarios considering diverse complexities.

### Realworld case study

This section illustrates how the proposed methodology was used in a real-world case study of a transportation system in eastern Switzerland, which includes roads and bridges vulnerable to flooding, mainly from the Rhine River. It begins with an overview of the case study and proceeds to detail the system representation, encompassing the input variables considered in the risk assessment, system outputs of interest, the simulation model used to predict outputs based on inputs, and the considered risk measure. Following this, the results of the reference risk assessment are outlined. Subsequently, candidate stress tests, defined using input variables, are introduced, and the results from the proposed methodology are presented, indicating the priority of stress tests to be executed using simulations.

### Transport system

**Fig. 8:**
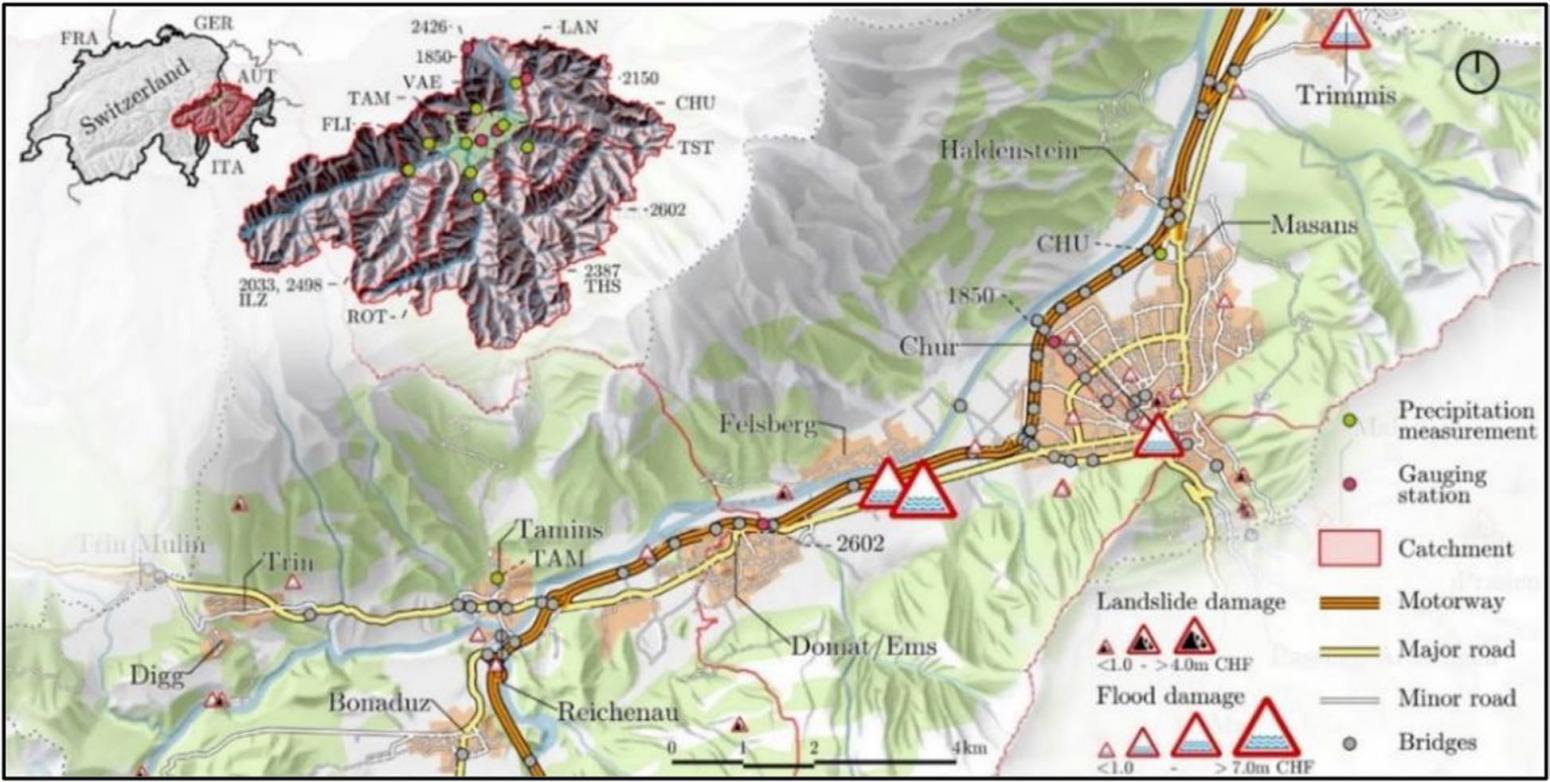
The studied transport network in Chur, Switzerland, adopted from Hackl et al. [20]

The transportation network is situated in the Chur region in the Canton of Grisons in Switzerland, as depicted in Fig. 8. It serves as a crucial transportation hub in eastern Switzerland, particularly for the transport of goods between the south and north of the Alps. The network comprises 605 km of roadways, of which 51 km are national motorways, and 121 bridges, including 18 bridges on the river and hence prone to scouring. The area lies in the Rhine Valley, between Trin and Trimmis, and has a history of flooding in the Rhine River [28]. Some of the historical flood events, along with the extent of their induced physical damage are displayed in Fig. 8. Two streams of water, namely the Anterior Rhine and Posterior Rhine, flow into the region from the southwest. Their discharge is respectively recorded at Ilanz (No. 2033, 2498) and Fürstenau (No. 2387) gauging stations. The two streams have a confluence at Reichenau, from where the river is called the Rhine River, which flows out of the region from the northeast. The discharge of the Rhine River is recorded at the Domat (No. 2602) gauging station. This area has been extensively investigated by several studies for risk assessment [20–22, 30].

### System representation

**Fig. 9:**
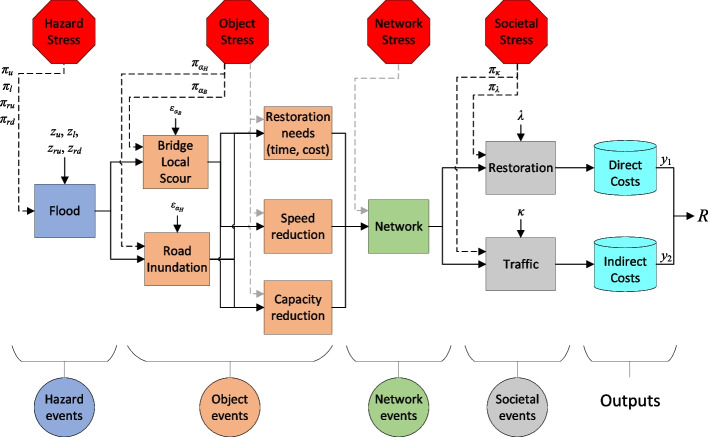
System representation: input random variables, simulation model, system outputs, and candidate stress tests (red hexagons). Notation is indicated in Table 3

Figure 9 shows the system representation for the studied transport system. The system is represented by four subsequent categories of events (circles) and their corresponding models (rectangles) each representing the behavior of one part of the system. It begins with hazard events that focus on predicting flood occurrences and generating spatial inundation maps. Object events predict flooding impacts on roads and bridges through models estimating physical damage, functionality loss, and the time and cost required for restoration activities. Network events predict the overall network performance by aggregating component behaviors in terms of drivable routes. Finally, societal events encompass models estimating the time and costs associated with restoration activities, as well as models predicting the state of services provided by the infrastructure, such as traffic flow and connectivity. Solid arrows entering the models from the left indicate how outputs of one model serve as inputs for others, and those entering from the top represent specific input variables for each model. For brevity, however, only those input variables considered in the stress test analysis are shown. The cylindrical boxes represent system outputs, including direct and indirect costs, introduced subsequently. For a detailed description of models, complete lists of input variables, and indicators used to monetize indirect consequences, please refer to Hackl et al. [20].

According to the stress testing methodology, stress tests can be defined on each part of the system, as represented by red hexagons in Fig. 9, including hazard, object, network, and societal stress tests in this case. The dashed lines represent the imposed conditions of each stress test. The black dashed lines, contrary to those in grey, indicate stress tests considered in the case study, along with their name.

### Input variables

In this section, input variables and their corresponding models that are subjected to stress testing are introduced. Table 4 shows a summary of input variables, together with their PDF, as well as the system outputs of interest and the considered risk measures, all described subsequently.

**Table 4:** Input variables considered for stress testing, system outputs, and risk measures

	Name	Description	Characteristic
Input variables	*z*_*u*_	Volume of floodwater in the upper-Rhine River for a 100 yr flood event	*N*(8504, 586^2^)
	*z*_*l*_	Volume of floodwater in the lower-Rhine River for a 100 yr flood event	*N*(14,914, 2083^2^)
	*z*_*ru*_	Volume of floodwater in the upstream of Rhine River for a 100 yr flood event	*N*(33,449, 3197^2^)
	*z*_*rd*_	Volume of floodwater in the downstream of Rhine River for a 100 yr flood event	*LN*(10.13, 0.20)
	*ε*_*αB*_	Uncertainty in the median fragility parameters of all damage states for bridges	*U*(0.25, 1.75)
	*ε*_*αH*_	Uncertainty in the median fragility parameters of all damage states for highways	*U*(0.25, 1.75)
	*λ*	Number of restoration teams available after the hazard event	*BN*(10, 0.5)
	*κ*	Average hourly aggregate traffic flow within the network	*N*(17,985, 3597^2^)
Outputs	*y*_1_	Direct costs of restoration activities	‒
	*y*_2_	Indirect costs due to disruption to the provided service by the network	‒
Risk measure	*R*	Sum of the mean values of the direct and indirect costs	$R = \overline{y}_{1} + \overline{y}_{2}$

**Fig. 10:**
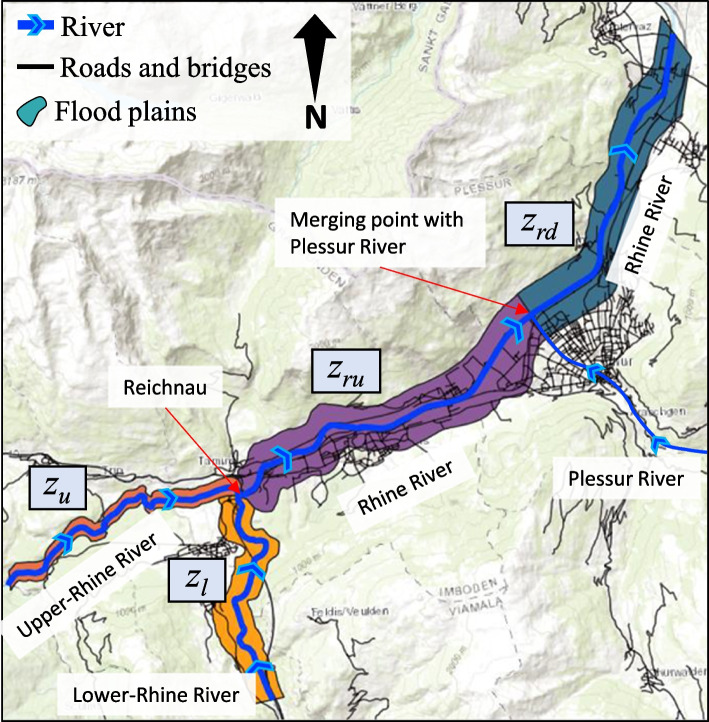
Four sub-areas and their corresponding variables capturing the volume of floodwater

The first four input variables represent the volume of flood water, in cubic meter, in four sub-areas as delineated in Fig. 10. These include Upper-Rhine River, Lower-Rhine River, upstream part of the Rhine River from Reichnau to where Plessur River merges with the Rhine, and the downstream part of the Rhine River from the Plessur river merging point to where the Rhine flows out of the boundaries of the region of study. The volume of floodwater in these four areas are respectively named as *z*_*u*_, *z*_*l*_, *z*_*ru*_, and *z*_*rd*_. These random variables serve as input to the flood model, which generates the spatial field of inundation depth over the entire area, considering its topography as given by the region’s digital terrain model (DTM).

Previous studies on this area have modelled the uncertainty associated with these variables for different return periods of flooding [20–22]. For risk assessment and stress testing in the case study, flood events with 100-year return period are considered, placing the focus of this study on PDF of these variables for 100-yr flood events, which are given in Table 4. The choice of 100-yr flood events is based on the common flood scenarios for risk assessment in Switzerland [17], although one can choose another intensity of events for risk assessment.

Following the flood model, damage models predict the damage state (DS) of roads and bridges. These models feature fragility curves for bridges and roads, which can be formulated by the following equation:

7 $$p\left( {DS \ge ds_{i} \left| {IM} \right.} \right) = \Phi \left( {\frac{{\ln \left( {IM} \right) - \alpha_{i} }}{{\beta_{i} }}} \right)$$

where *ds*_*i*_ is the damage state *i*, IM is the intensity measure, here the inundation depth, *α*_*i*_ is the median fragility level, i.e., the intensity level at which the probability of damage state *ds*_*i*_ is 50%, and *β*_*i*_ is the variability in the fragility curve for *ds*_*i*_. For the case study, there are two types of bridges, i.e., one-pier and two-pier, and three types of roads, i.e., highway, major, and minor. For each asset type, three damage states have been defined, namely, slight, moderate, and severe. For each asset type, and for each damage state, *α* and *β* are quantified. For a more detailed description of damage states, and their *α* and *β* parameters, please refer to Hackl et al. [20].

The parameters *α* and *β* are inherently uncertain due to limited data, modeling assumptions, and variability in infrastructure response to hazard intensities [8]. This uncertainty results in a bandwidth around the fragility curves, representing the range of potential damage outcomes for a given hazard intensity. Within this bandwidth, variability exists in the predicted probability of damage, leading to scenarios where infrastructure performance may deviate from the expected prediction by the aggregate fragility model. In some scenarios, infrastructure performs better than expected, resulting in lower damage levels, while in some, it performs worse, leading to higher damage.

In this study, particular attention is directed towards considering the uncertainty in the *α* parameter of fragility curves, specifically focusing on highways and bridges. The uncertainty in these parameters, particularly in scenarios pertaining to roads and bridges subject to flooding, has been investigated previously [21]. To model this uncertainty in the current study, certain assumptions and simplifications are made, although future research can be dedicated to refining this uncertainty.

This uncertainty for bridges and highways is denoted as *ε*_*αB*_ and *ε*_*αH*_, respectively. A uniform distribution between 0.25 and 1.75 is used to probabilistically model these uncertainties, hence, *ε*_*α*_ ~ *U*(0.25, 1.75). Therefore, in each generated scenario, the *α* parameter for each damage state can be modelled as $\alpha =\widetilde{\alpha }\times {\varepsilon }_{\alpha }$, where $\widetilde{\alpha }$ is the expected value of the parameter suggested by previous deterministic studies. If the estimated *α* exceeds $\widetilde{\alpha }$ it signifies situations where the probability of experiencing more severe damages is reduced, whereas scenarios with *α* values lower than expected indicate higher vulnerability to flooding.

The next uncertainty considered arises from the variability in the availability of post-hazard restoration resources. Specifically, the focus is on the number of restoration teams and contractors capable of initiating and executing recovery efforts following a flood event. While there is a general understanding of the expected available resources, various factors contribute to fluctuations, e.g., accessibility, logistical constraints and workforce availability. These factors introduce unpredictability, resulting in situations where the actual availability of resources exceeds or falls short of the initial expectations. This variability highlights the complexity of disaster recovery, where diverse factors interact to shape post-hazard resource allocation.

Previous studies on the area considered the expected number of restoration teams to be five, with no indication of its uncertainty. Considering this, and to model the uncertainty in this variable, a Binomial PDF is used to model this variable such that the expected value would be five. The considered Binomial PDF for this variable, as given in Table 4, is *λ* ~ *BN*(*n* = 10, *p* = 0.5), hence, *μ*_*λ*_ = 5 and *σ*_*λ*_ = 2.5.

The next uncertainty considered is related to the variability associated with the traffic flow. This random variable captures the fluctuations in the number of travelling vehicles in the network, which may deviate from expected levels due to several factors, such as weather conditions or shifts in travel patterns. Previous studies on the area considered a deterministic value for this variable, representing the expected hourly traffic flow in the network to be 17,985 vehicles. To model the uncertainty in this variable, a normal PDF with a mean of 17,985 and a 0.2 coefficient of variation was considered, resulting in a standard deviation of 3,597., i.e., *κ* ~ *N*(17,985, 3597^2^).

### System outputs and risk measures

The two outputs considered here are direct costs, denoted as *y*_1_, and indirect costs, denoted as *y*_2_. Direct costs entail expenses associated with restoration interventions, including inspection and repair costs for damaged infrastructure. Indirect costs encompass factors such as increased travel time, changes in vehicle operation such as fuel consumption, and missed trips due to connectivity loss. Given these system outputs, and consistent with previous studies on this area, the sum of the mean values of the direct and indirect costs is considered as the risk measure, *R*. In essence, *R* shows the expected total costs of a 100-yr flood event.

To assess the acceptability of risks using cost-based risk measures, previous studies suggested thresholds based on proxies reflecting the economic capacity of the region, such as its gross domestic product (GDP). The decision on selecting an appropriate threshold and the determination of what constitutes an acceptable risk depend on stakeholder opinions and the capability of a country and centralized civil protection authorities to support further actions and implement risk-reducing measures based on the level of urgency and severity. It is to say that the objective of this study is not assessing whether risks and stress tests are acceptable, rather prioritizing them for more detailed assessment. Nevertheless, for illustrative purposes and to show the capabilities of the proposed methodology, in line with previous studies on this area, 1% of the GDP of the canton of Grisons was considered as the risk threshold. Based on the region’s GDP of 14.4 billion CHF in 2016 [18], the threshold is set at 144 million CHF.

### Reference risk assessment

For reference risk assessment, an analysis period of one year is considered, assuming that only one flood event occurs. For the reference risk assessment, *N* = 1′500 random scenarios were generated. Each scenario is a random realization of the system, starting from a random spatial field of inundation for a 100-year flood event, to the extent of damage to roads and bridges, to their functionality loss in terms of reduction in speed and capacity, to the state of traffic flow within the network, to an estimation of the time and cost required for restoration. This results in the estimation of the ensuing direct costs (*y*_1_) and indirect costs (*y*_2_) for each scenario.

**Fig. 11:**
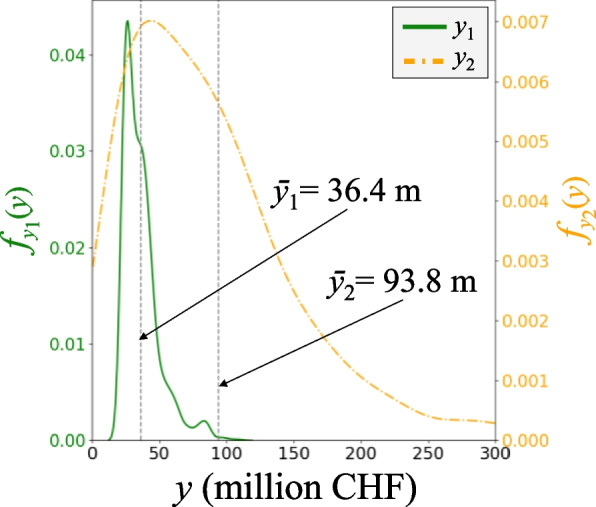
PDF of system outputs using 1500 simulations

Figure 11 shows the empirical PDF of the system outputs *y*_1_ and *y*_2_. The mean values of these variables are also shown on their respective curves, suggesting that indirect costs are significantly larger than direct costs. The difference is even more pronounced in the extremes, with indirect costs exceeding 6 m CHF in some scenarios, as opposed to 1 m CHF for direct costs. Accordingly, the considered risk measure is calculated as *R* = 130.2 m. CHF. This risk essentially shows the expected overall costs that the investigated transport system has to incur under a 100-yr flood event.

### Stress test prioritization

Table 5 presents the list of stress tests considered, together with their relevant variable. These stress tests encompass a wide range of scenarios including more extreme flood events (*π*_*u*_, *π*_*l*_, *π*_*ru*_, *π*_*rd*_), more vulnerable assets (*π*_*αB*_, *π*_*αH*_), higher traffic demand (*π*_*λ*_), and fewer restoration teams (*π*_*κ*_). A similar approach as the one used in the numerical example is used to model these stress tests. That is, for each stress test, the mean parameter of the PDF of the relevant variable is shifted by a measure, denoted as *δ*_*π*_, such that the likelihood of having unfavorable values would be higher than the reference. In this change, other parameters of the PDF are kept unchanged. Therefore, the imposed condition by all stress tests can be formulated as *μ*|*π* = *μ* + *δ*_*π*_.

**Table 5:** Stress tests for the case study, their target variable, and their description

Stress test	Target variable	Description
*π*_*u*_	*z*_*u*_	Increase in the extent of flooding in the Upper Rhine River
*π*_*l*_	*z*_*l*_	Increase in the extent of flooding in the Lower Rhine River
*π*_*ru*_	*z*_*ru*_	Increase in the extent of flooding in the upstream part of the Rhine River
*π*_*rd*_	*z*_*rd*_	Increase in the extent of flooding in the downstream part of the Rhine River
*π*_*αB*_	*ε*_*αB*_	Increase in the vulnerability of bridges subject to local scouring
*π*_*αH*_	*ε*_*αH*_	Increase in the vulnerability of highways subject to inundation
*π*_*λ*_	*λ*	Having less restoration teams after the flood event
*π*_*κ*_	*κ*	Having higher traffic flow in the network at the time of flooding

To perform the stress perturbation analysis, a range of [‒7.5%, 15%] with 2.5% increments was considered as the relative change in the mean of each variable. This resulted in conducting the proposed resampling approach for 80 iterations (10 increments for 8 variables). Obtaining more accurate results for these 80 scenarios by explicitly conducting simulations would have taken 56 weeks for the case study, with each assessment taking one week of computation using a server equipped with dual 10-core Intel Xeon E5 - 2690v2 processors running at 3.0 GHz and 384 GB of DDR2 RAM, operating on Ubuntu 64-bit.

**Fig. 12:**
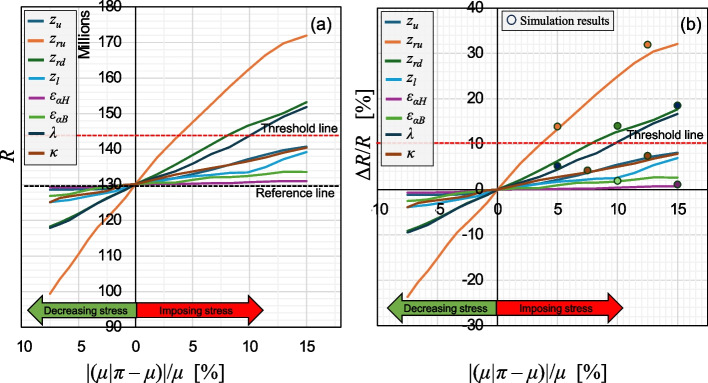
Stress perturbation analysis: a) risk measure vs. change in the mean of the variable, b) relative change in the risk measure vs. change in the mean of the variable

Figure 12 shows the results of the stress perturbation analysis. Figure 12a and b show the estimated values of *R* and their relative change against the relative change in the mean of the variables, respectively. It suggests that under a similar change in the mean of the variables, the extent of flooding in the upstream and downstream parts of the Rhine River has a larger impact on risks, followed by the traffic flow and number of available restoration teams. Additionally, the performance of the bridges has a larger impact on risks than highway sections. Figure 12b also shows simulation results from conducting some selected stress tests, suggesting a relatively close alignment, with a maximum error of 7.4%.

**Fig. 13:**
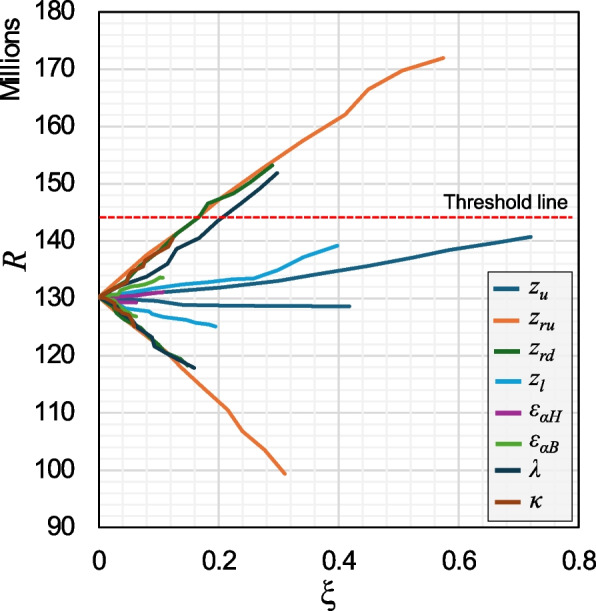
Risk measures vs. stress level for the 8 investigated stress tests

Figure 13 shows the values of the risk measure against to the stress level *ξ*. Note that, even though the range of change to the mean was similar for all variables, the corresponding stress level varies significantly due to its dependence on the entire PDF.

Table 6 presents the calculated rank measures for the considered stress tests for both cases of imposing stress and decreasing stress. Note that, to achieve consistency in the stress level for various stress tests, rank measures are calculated based on a common range of stress level. For both cases of imposing and decreasing stress, stress tests concerning the extent of flooding in the two sections of the Rhine River were identified as the most important, with ${I}_{ru}^{s}=$ 0.37 and ${I}_{rd}^{s}=$ 0.22. An interesting observation is that imposing stress on available restoration resources (${I}_{\kappa }^{s}=$ 0.22) shows larger impact than travel demand (${I}_{\lambda }^{s}=$ 0.11); however, when considering their importance under stress reduction, their ranking is reversed (${I}_{\kappa }^{p}=$ 0.13 and ${I}_{\lambda }^{p}=$ 0.16). This can provide insights such as the need for decision makers to ensure that the availability of restoration resources does not fall below planned levels, for instance, by increasing the reliability of existing resources rather than necessarily increasing their quantity, considering budgetary constraints. Similar conclusions can be drawn for other trade-offs between target variables and their importance, which is evidence of the significance of this paper in assisting infrastructure managers to identify which stress tests have larger potential to increase risks, and therefore require computational resources.

**Table 6:** Rank measures for the stress tests in the case study

Stress test	Imposing stress	Decreasing stress
*π*_*u*_	${I}_{u}^{s}$= 0.005	${I}_{u}^{p}$= 0.003
*π*_*l*_	${I}_{l}^{s}$= 0.02	${I}_{l}^{p}$= 0.06
*π*_*ru*_	${I}_{ru}^{s}$= 0.37	${I}_{ru}^{p}$= 0.31
*π*_*rd*_	${I}_{rd}^{s}$= 0.22	${I}_{rd}^{p}$= 0.26
*π*_*αB*_	${I}_{{\alpha }_{B}}^{s}$= 0.04	${I}_{{\alpha }_{B}}^{p}$= 0.06
*π*_*αH*_	${I}_{{\alpha }_{H}}^{s}$= 0.01	${I}_{{\alpha }_{H}}^{p}$= 0.02
*π*_*λ*_	${I}_{\lambda }^{s}$= 0.11	${I}_{\lambda }^{p}$= 0.16
*π*_*κ*_	${I}_{\kappa }^{s}$= 0.22	${I}_{\kappa }^{p}$= 0.13

Another insight that can be gained from the presented results is by observing the risk threshold line in Fig. 12. It helps identify the level of stress or the degree of change to the mean of each variable that can cause risk to surpass the threshold. For example, for stress tests *π*_*ru*_, *π*_*rd*_, and *π*_*λ*_, increases of 3.5%, 7.5%, and 9.6% in the mean of their relevant variables, respectively, can result in exceeding risk thresholds. This information can help decision makers be prepared and, if needed, plan for interventions to prevent these variables from exceeding their acceptable boundaries. This insight can be readily obtained using the proposed methodology, as opposed to spending a significant amount of time running simulations for these stress tests.

## Discussion

This section provides a discussion on factors, besides sample size as discussed earlier, that influence the performance of the proposed methodology in terms of accurate estimation of risks. It is noted that the goal of the proposed methodology is to provide relatively accurate risk estimations without simulations, but otherwise, one can achieve more accurate results using detailed simulations, yet at the cost of computation.

The reliability of any risk assessment, including those using the proposed methodology, depends on the quality of the input distributions, which are often based on a combination of expert judgment and available data. In many practical cases, especially where data is limited, some level of bias or uncertainty is unavoidable. Stress testing, nonetheless, remains valuable even with limited data, as long as the results are interpreted with care. It can also help identify where improved data or further analysis may be most useful. In such cases, efforts to expand data collection or refine input assumptions over time can help strengthen the reliability of assessments.

Referring to Fig. 12b, an interesting observation is that in all scenarios, simulation results are larger than estimations. This highlights an important factor that significantly impacts the accuracy of estimations, which is the sensitivity of the system output to changes in the extreme values of input variables. Extreme values have a higher likelihood of occurrence under stressed conditions compared to the reference PDF. When the system exhibits high sensitivity to ranges of values of a variable that are more prevalent in the stressed PDF, estimation results can be lower than simulation results because those extreme values may not be well represented in the reference sample. This is because of their relatively lower likelihood in the reference PDF compared to the stressed situation. This emphasizes the importance of having reference simulations accurately capture and represent these extreme ranges, otherwise, they can lead to biased risk estimates under stressed conditions.

The next factor is how inclusive the reference sample is in terms of covering the range of values in the stressed PDF or, in other words, how well the stressed PDF can be created using the sample generated using the reference PDF. For example, in a case where the stressed PDF is so different from the reference PDF that the range it is defined on has little to no overlap with the reference, it is impossible to estimate risks under stressed situations using the resampling approach. In these situations, it is advised to explicitly conduct stress tests using simulations. However, performing a stress perturbation analysis for lower stress levels can provide an indication of the potential impact of the stress test. As the overlap between the PDFs decreases, the effectiveness of the resampling approach in representing the stressed PDF and accurately estimating risks diminishes.

The performance of certain analyses is influenced by whether the PDF is bounded or unbounded. When dealing with bounded PDFs, it may be feasible to have resampled sets replicate the stressed PDFs with sufficient reference simulations. Yet, in cases where the PDF is unbounded, the effectiveness of resampling diminishes, particularly when the stressed PDF significantly deviates from the reference. In this case, it is advised to keep the imposed stress levels for the stress perturbation analysis under a certain threshold and ensure that there are enough simulations that have acceptable coverage of the potential range of values in the stressed PDF. It should also be emphasized that stress tests can evaluate scenarios beyond those captured by the reference PDFs, such as hypothetical increases in the magnitude or frequency of certain variables, pushing distributions beyond initial bounds or significantly altering their shapes. Hence, stress testing provides a unified framework to systematically explore adverse scenarios—both within and beyond existing distributions—to comprehensively assess potential risks.

The next factor lies in the potential disproportionate impact of sample points with high weights on the estimation process. Sample points with high weights, characterized by a high ratio between the stressed and reference PDFs, tend to be selected more frequently during resampling. These high-weight sample points may represent regions of the distribution where the stressed PDF is significantly heavier than the reference PDF. Consequently, the resampling process may not be able to accurately capture the nuances of the stressed PDF, thereby potentially leading to biased risk estimates. To avoid misinterpretations of the results and ensure that the results are not unduly influenced by high weighted samples, it is advised to validate results for some scenarios using simulations.

As previously mentioned, the current implementation of the proposed methodology is applicable only to single-stressor stress tests, which affect the PDF of a single variable. It has not yet been extended to multi-stressor stress tests that impact multiple variables, either by changing their PDFs or their correlations. Nonetheless, the methodology for multi-stressor scenarios would follow the same principles. Instead of using the PDF of one variable to calculate sample point weights, the joint PDF of the affected variables would be used. Extending the use of the proposed methodology to all possible stress test scenarios is a topic for future research.

A limitation of the proposed methodology arises when dealing with deterministic parameters—those with fixed values in the reference assessment. If a stress test scenario involves changing these deterministic parameters, the proposed resampling approach cannot be applied. Under these circumstances, simulations must be run to evaluate the stress tests. For the methodology to be effective, all variables need to be modeled probabilistically. This probabilistic modeling allows for the calculation of sample point weights and the accurate assessment of risk under stressed conditions.

The rank measure proposed in this paper is based on the premise that stress tests have been identified, yet their specifics are not yet defined. Therefore, a stress perturbation analysis is conducted considering various stress levels to provide a proxy to gauge the potential impact of the stress tests on risks. However, if specific stress levels are predefined for the stress tests, one can estimate the risks directly under those scenarios and rank them based on their relative increase in risks and whether they cause risks to exceed thresholds.

An important consideration regarding stress test scenarios is that assigning explicit probabilities to these scenarios is often infeasible due to inherent deep uncertainties. Certain scenarios commonly used in stress testing involve plausible yet uncertain futures, making probabilistic quantification or relative ranking impractical or impossible. For instance, a common application of stress testing is the evaluation of system performance and risks under various climate change projections, specifically the Representative Concentration Pathways (RCPs) such as RCP 2.6, RCP 4.5, or RCP 8.5. These scenarios represent distinct, plausible future states, yet assigning precise probabilities is infeasible. This aligns closely with the notion of deep uncertainty [42], where neither probability distributions nor relative likelihood rankings among certain scenarios can be confidently established. Given this inherent uncertainty, the practical focus of stress testing shifts away from assigning exact probabilities toward evaluating system vulnerabilities and identifying conditions under which risks may disproportionately increase. The proposed methodology specifically addresses this need by enabling decision-makers to efficiently prioritize scenarios based on their relative impacts without relying on computationally intensive simulations.

It should be noted that the proposed methodology does not provide guidance on the plausibility of scenarios, how stress test scenarios should be initially selected, the specific conditions of each scenario, or the number of scenarios to consider. The systematic selection and specification of stress test scenarios remains an open research question. Rather, the proposed methodology operates under the assumption that candidate stress tests have already been identified. Given such a predefined set of stress tests, the methodology offers a computationally efficient screening analysis of a wide range of scenarios, including extreme or exploratory ones, to determine which ones potentially have the most significant impact on risks, thereby helping decision-makers prioritize scenarios for detailed simulation-based investigations. Whether a scenario—regardless of how extreme it is or how likely it may be—is relevant, plausible, or worthy of consideration remains the responsibility of decision-makers and domain experts, and lies beyond the scope of the proposed methodology. When specifying the exact scenarios of a stress test, it is important that decision-makers carefully consider their plausibility taking into account various practical constraints—such as infrastructure limitations, regulatory conditions, or operational feasibility.

## Conclusion and Outlook

The proposed methodology offers a computation-free approach for assessing the impact of stress tests and prioritizing them for further evaluation using simulations. The primary aim of stress testing is to define specific scenarios, evaluate risks, and assess their acceptability. However, defining these scenarios for the wide range of potential stress tests is challenging because of the tedious analyses required. Therefore, in the absence of specified scenarios, the proposed methodology employs a proxy to identify stress tests that can potentially lead to higher increases in risks. The proxy is based on the relative increase in risks with respect to the change to the Probability Density Function (PDF) of the variable affected by the stress test. With the challenge of numerous stress tests, each consuming considerable computational resources to be assessed using simulations, this methodology enables a rapid screening analysis to pinpoint stress tests that could potentially elevate risks beyond acceptable thresholds.

The proposed methodology entails a novel implementation of bootstrap resampling and importance sampling approaches to estimate the impact of stress tests. It receives the simulation results of an initial reference risk assessment, together with the imposed changes of each stress test to the PDF of its corresponding variable and estimates risks, eliminating the tedious task of additional simulations. This is achieved by selecting a resampled set of the reference sample such that it replicates samples generated under stressed conditions. The computational efficiency of the proposed methodology enables repeated analyses across various intensity levels of stress tests, referred to as stress perturbation analysis. This capability allows for a more comprehensive exploration of the system risk profile, as decision-makers can systematically assess how different stress levels impact risk, and which stress level can result in risks surpassing thresholds, without incurring additional computational costs.

The performance of the proposed methodology in accurately estimating risks under stress tests depends on various factors. First is the number of simulations conducted for reference risk assessment. Besides, sensitivity of system outputs to extreme input values can lead to over- or under-estimation of risks. Another factor is the inclusiveness of the reference sample in covering the stressed PDFs. Having sample points that are over proportionally more likely to occur under stress conditions can also lead to biased risk estimates.

An important consideration for assessing the reliability of estimates under stress scenarios is the divergence between the reference and stressed distributions. When this divergence is large—particularly if the stress scenario shifts probability mass into regions poorly represented by the reference sample—the effectiveness of the resampling-based estimation may decrease. In such cases, measures such as the Kullback–Leibler (KL) divergence [35] or the Effective Sample Size (ESS) [29] can be useful tools to evaluate the adequacy of the sample. KL divergence quantifies the difference between two probability distributions, while ESS indicates the number of samples that effectively contribute to the estimate after importance weighting. When either metric suggests poor overlap or sample degeneracy, caution should be exercised in interpreting results. Although these diagnostic tools are not integrated into the current implementation, their incorporation represents a promising direction for future refinement of the methodology.

The proposed methodology has some limitations that suggest avenues for future research. It mainly applies to single-variable stress tests, limiting its scope for scenarios involving multiple stressors, i.e., those that affect the PDF of multiple variables or their correlation simultaneously. Future work could focus on expanding the methodology to handle such scenarios, which are common in real-world systems, e.g., increased intensity of flood events combined with higher traffic flow. Another promising avenue for future research lies in establishing robustness criteria to evaluate the reliability of the proposed methodology's results. These criteria could include thresholds for percentage of coverage of the stressed PDF by the reference sample or weights of sample points. Additionally, further research could focus on developing precise criteria for defining specific stress test scenarios, ensuring they are tailored to accurately assess and enhance system resilience under various conditions.

## Data Availability

Data is provided within the manuscript or supplementary information files.
